# Reading between the whines: human perceptions and beliefs about animal emotions predict how people would intervene with cats and dogs showing challenging behaviors

**DOI:** 10.3389/fpsyg.2026.1857104

**Published:** 2026-07-15

**Authors:** Anna E. Holloway, Michael A. Kisley

**Affiliations:** Psychology Department, University of Colorado Colorado Springs, Colorado Springs, CO, United States

**Keywords:** anger, animal emotion, beliefs about animal emotion, companion animal, fear, intervention, perception

## Abstract

Previous research has shown that differences in people’s experiences with and beliefs about animals—in addition to contextual factors—influence how they perceive pet emotions, which is associated with animal welfare. The present study examined whether behavioral intensity, beliefs about animal emotions, experience with animals, and perceived emotion predict how a person would intervene when cats and dogs exhibit defensive behaviors. A U.S. sample (*N* = 194) completed vignette-based tasks manipulating species (dog vs. cat) and behavioral intensity (low vs. high). Participants rated perceived animal fear and anger and indicated their likelihood of utilizing each of six intervention strategies. Measures of beliefs about animal emotions and animal-related experience were also collected. High-intensity behaviors increased both perceived emotional intensity and the likelihood of intervention. Fear was attributed more than anger overall, and perceptions of anger increased with behavioral intensity. Perceived fear predicted lower-impact responses (e.g., relocation). Perceived anger, however, predicted higher-impact interventions (e.g., relinquishment and spanking). Beliefs about the moral relevance of animal emotions predicted a reduced likelihood of using the same disruptive or aversive interventions. These findings extend prior research by showing that emotional perceptions and participant characteristics shape responses to companion animal behavior, with implications for animal welfare and human–animal relationships.

## Introduction

Companion animals are extremely popular in many parts of the world, with dogs and cats representing the most common companion species across the 22 countries surveyed (Health for Animals Global Animal Health Association, 2022). In the United States alone, 51% of households have a dog and 37% of households have a cat; an estimated 94 million American households have one or both companion species (American Pet Products Association (APPA), 2025). Pet behavioral concerns are common (Buller and Ballantyne, 2020), tend to be closely related to perceived pet–owner relationship quality (Hoffman et al., 2013), and are also a leading reason for the relinquishment of animals to shelters (Kisley et al., 2024a; Powell et al., 2022). When companion animals display challenging or defensive behaviors, people may respond in a variety of ways, and these responses are not equivalent in their potential consequences for the animal. For example, relinquishment to a shelter and the use of aversive forms of punishment can be stressful or otherwise disruptive (Dybdall et al., 2007; Guilherme Fernandes et al., 2017; Vieira de Castro et al., 2020), whereas some management- and treatment-oriented approaches may address behavioral concerns with fewer negative consequences (Argüelles et al., 2023; Lopes et al., 2022). Therefore, understanding what leads people to favor one response over another has practical significance for companion animal care. One factor that may shape these choices is how people interpret an animal’s underlying emotional state.

Since humans and their companion dogs and cats do not have a shared spoken language, an important means of communication is the emotional interpretations of behavior and vocalizations (Norrthon and Nilsson, 2025; Barklam and Felisberti, 2024). It is important to study these common human–animal interactions to better understand what might influence one’s perception of the emotional state of an animal, thereby improving one’s ability to understand and interact with one’s companion. In addition, interpreting an animal’s behavior to infer its underlying emotion has been shown to be a non-invasive method of assessing animal welfare and is an especially useful technique when tools used to measure animals’ physiological markers associated with their welfare (e.g., cortisol levels, heart rate, etc.) are unavailable (Czycholl et al., 2026; de Winkel et al., 2024). That said, observing only an animal’s behavior to infer its underlying emotion is not always sufficient or accurate, and the addition of external information, including what the animal is reacting to, appears to increase a person’s ability to perceive the animal’s underlying emotion (Molinaro and Wynne, 2025). However, even when participants are shown the same photos of animal behavioral expression, they do not always attribute the same emotion to each animal (Bouma et al., 2023). These discrepancies in the attribution of animal emotions suggest that people’s judgments are shaped by many factors, including the amount of situational context provided alongside the behavior, underscoring the need to better understand what influences perceptions of animal behavior in human–animal interactions.

In addition to accounting for the influence of animal behavior and situational context, variation in people’s understanding of animal emotions should also be investigated, as this has been shown to be associated with the human–animal relationship. For example, the greater the extent to which one believes that their working equid is capable of feeling emotions, the higher the measured objective welfare of that animal (Haddy et al., 2025). Another study found that beliefs about the emotional capacities of stingrays predict a greater likelihood of voting for (and donating to) shark and stingray conservation efforts (Hancock et al., 2023). In addition, it has been shown that cross-cultural differences in beliefs about the emotional experience of non-human animals are associated with differences in the way animals are treated across cultures (Tamioso et al., 2018). Hica et al. (2026) found that when pet owners view their companion animal as an emotionally significant member of the household, there is an increase in affiliative behaviors (e.g., petting, talking to the pet, and hugging), make-up behaviors after conflict, and perceived emotional support. Taken together, these studies suggest that the way people think about animals’ emotional capabilities influences their concern for and interaction with them. The present study aims to extend this area of research in a novel way by investigating whether people’s perceptions of which emotions an animal experiences, and how strongly, predict their preferred approach to intervening when a companion animal exhibits challenging behaviors.

Many participant characteristics, including one’s prior experience with animals, have been shown to be associated with people’s perceptions of animal emotions. For example, children’s ability to attribute a dog’s underlying emotion varies widely due to several variables, including the cultural context in which the child was raised (Amici et al., 2019) and the child’s age, such that their perception accuracy seems to increase as they grow older (Lakestani et al., 2014). This can be potentially dangerous when children misperceive anger as happiness, unknowingly putting themselves at risk of being bitten (Aldridge and Rose, 2019). In adults, pet ownership significantly improves the accuracy of perceiving dog emotions (Hawkins et al., 2021; Lakestani et al., 2014). Relatedly, Wan et al. (2012) found that greater experience with dogs predicts the identification of fear as the primary emotion underlying a dog’s behavioral display. Studies have shown that fear is the least correctly attributed animal emotion overall (Amici et al., 2019; Catalán et al., 2020; Guo et al., 2024) and that companion animal ownership increases one’s ability to perceive fear in dogs. In addition to animal ownership, one’s professional experience with animals can also affect their interpretation of dog (Catalán et al., 2020) and cat emotions (de Mouzon et al., 2023). Individual differences in empathy for animals have also been shown to predict sensitivity to negative emotions communicated through dog, but not cat, vocalizations (Barklam and Felisberti, 2024). Overall, the available literature supports the idea that people’s perceptions of animal emotions vary from one individual to another, and these variations are associated with certain participant characteristics and important human–animal outcomes.

This study includes novel features while being informed by and grounded in previous literature on the perception of animal emotions. Previous studies on the attribution and perception of companion animal emotions have utilized audio (Barklam and Felisberti, 2024), video (Lakestani et al., 2014; Demirbas et al., 2016; Guo et al., 2024; Wan et al., 2012), still photographs (Bouma et al., 2023; Amici et al., 2019), or various combinations of these stimulus types (Aldridge and Rose, 2019; Molinaro and Wynne, 2025; Catalán et al., 2020; de Mouzon et al., 2023). With one exception we found, which aimed to investigate attitudes toward euthanizing horses (Bell and Rogers, 2021), the use of vignettes to control and manipulate behavioral variables has not been widely employed to study human perceptions of animal emotional states. In this study, we used written, standardized stimuli to present participants with an animal’s defensive behavior without any visual or auditory representations. Sheringham et al. (2021) noted that vignettes may reduce the possible influence of observational biases by standardizing the message into written words. Another study found that vignettes, compared to video, do not change the emotional impact of the overall message, supporting the idea that vignettes are an appropriate way to present participants with an emotional display (Smets et al., 2025). For perceived emotions, we focused on fear and anger because these are the most frequent motivations underlying an animal’s defensive behavior (Bowen and Heath, 2005, Chapters 11 and 16; Riemer et al., 2021), yet the line between these two emotions is often difficult for humans to distinguish for both cats and dogs (Bouma et al., 2023; Guo et al., 2024; Hawkins et al., 2021). For this study, we did not attempt to assess the correctness of participant emotion ratings by comparing them to expert assessments (e.g., Bouma et al., 2023; Amici et al., 2019; Catalán et al., 2020; Guo et al., 2024; de Mouzon et al., 2023) because one’s construal of the world influences behavior regardless of objective accuracy (Wilson, 2022). We included measures of participants’ beliefs about the emotional capacity of animals because such pre-existing mindsets have been shown to predict attitudes toward animals (Haddy et al., 2025; Hancock et al., 2023; Tamioso et al., 2018). We also assessed participants’ beliefs about the moral relevance of animal emotions (Kisley, 2025) because such beliefs may influence whether individuals are more likely to engage in more disruptive interventions with their companion animals. Finally, previous experience with companion animals was incorporated in this study because of the extensive literature connecting this variable to the perception of animal emotions (Amici et al., 2019; Barklam and Felisberti, 2024; Catalán et al., 2020; Lakestani et al., 2014; Wan et al., 2012).

In this study, we tested the relationship between the intensity of a defensive behavior (low or high), species (cat or dog), participants’ perceptions of the animal’s emotions (fear and anger), and their pre-existing beliefs about animal emotions, as well as how all of these variables may predict the interventions that participants would be likely to employ to address challenging behaviors in their animals. To do so, two vignettes were presented to participants, one for each species type, in either the high- or low-intensity experimental condition. In the low-intensity condition, the cat[dog] stiffens, pulls back, and hisses[growls] at the stranger before backing away. In the high-intensity condition, the animal bares its teeth, pins its ears back, and stays close to the stranger; animals in this condition yowl[bark] at the stranger in addition to hissing[growling]. The physical behaviors and vocalizations described in the vignettes are common for both cats and dogs acting defensively (Riemer et al., 2021). After reading the vignettes, participants ranked the levels of both perceived fear and perceived anger as independent emotions, in addition to their likelihood of using six intervention techniques. Then, participants completed surveys and questionnaires measuring their experience with and responsibility for cats and dogs, their beliefs about animal emotions, and their implicit theories about animal emotions. These variables were then used to predict people’s likelihood of taking a range of corrective actions, from moving the animal to another room to surrendering the animal to a shelter. The main goal of this study was to examine how the emotion perceived to underlie defensive behavior, together with various participant characteristics, relates to human–animal interactions, particularly by shaping a person’s corrective response to an animal’s defensive behaviors.

## Methods

### Participants

Participants for the current study were recruited from Prolific, an online crowdsourcing site that connects research participants to studies for which they are eligible and has been shown to yield high-quality data (Peer et al., 2017; Peer et al., 2022). Eligible participants were at least 18 years old and currently residing in the US.

A total of 200 participants were recruited. A total of one participant failed at least one attention check (four attention checks were distributed throughout the survey: two were embedded within the Beliefs About Animal Emotions Scale (BAES) Qualtrics block, and one was embedded in each species’ exposure scale Qualtrics block) and was excluded. In addition, five participants who completed the survey were excluded for finishing in less than half of the median completion time, as recommended by Leiner (2019) to improve data quality. This yielded a final sample of 194 participants for the analyses. Participants’ age ranged from 21 to 78 years old (*M* = 46.95, *SD* = 13.29). The reported sex distribution was 55% female, 44% male, and 1% nonbinary. The ethnicity of participants was reported as 77% White, 8% Black, 6% Asian, 4% Hispanic or Latino, 3% Multiracial, and 2% Indigenous, Aboriginal, or First Nations. The distribution of the highest level of education completed was 1% with no high school diploma, 16% with a high school diploma or equivalent, 25% with some college degree, 43% with a bachelor’s degree or equivalent, 10% with a master’s degree or equivalent, and 4% with a doctorate or equivalent. This study received approval from the University of Colorado, Colorado Springs Institutional Review Board, and all participants provided informed consent.

### Materials and design

A mixed between- and within-subjects design was used to examine group and species differences in perceived emotions during a defensive behavior, as well as the likelihood of using different intervention techniques in response to the behavior. The between-subjects variable was the intensity of the defensive behavior, with two levels: low intensity and high intensity. The within-subjects variable was species, with two groups: cat and dog. Emotion type and intervention technique also served as within-subjects variables, as all participants rated the levels of anger and fear they perceived in both cat and dog vignettes, as well as their likelihood of utilizing each of six intervention techniques described below. Qualtrics’ randomization tool was used to evenly and randomly assign participants to one of the between-subjects conditions. For this purpose, four short vignettes were created: two for dogs and two for cats, corresponding to low- and high-intensity conditions; all four finalized vignettes are shown in Table 1. To develop these materials, ChatGPT-5 (OpenAI, 2025) was prompted to generate two vignettes that could be easily adapted to refer to a cat or a dog. These vignettes were then edited to create the low-intensity condition and used as a framework for creating the high-intensity vignettes. The defensive behaviors presented in the vignettes did not include physical contact (i.e., biting and hitting) but consisted of vocal and visual behaviors that were consistent across species (i.e., [low-intensity] pulling back, [high-intensity] pinning ears back, and baring teeth), as well as species-specific behaviors (i.e., [low- and high-intensity] hissing vs. growling and [high-intensity] yowling vs. barking). To increase the likelihood that participants would feel a connection with these imagined companion animals, the subject of each vignette was individualized with a name and was referred to throughout the vignette using he/him pronouns. The names included in the vignettes—Charlie, Milo, Max, and Ollie—appeared on the lists of the most popular male dog and cat names in 2024 (American Kennel Club Staff, 2024; The Dog People by Rover, 2024). In each vignette, participants were told they had “recently adopted” the cat or dog, in an attempt to reduce the amount of responsibility participants may assign themselves if they failed to correct the “problem” behavior when the animal was young. The use of the time frame “recently adopted” was intended to reduce potential confounding variables related to attachment. For example, the amount of time spent bonding with a companion animal may influence a guardian’s likelihood of excusing any defensive behaviors.

**Table 1:** Vignette stimuli.

Vignette intensity	Cat	Dog
Low-intensity	It’s late afternoon in the living room. You recently adopted a cat named Milo. A new visitor steps into the room and reaches toward Milo to say hello. Milo stiffens as he pulls back and hisses at the visitor. The interaction lasts only a few seconds, and the visitor backs away.	It is early afternoon on a Monday in the dining room. You recently adopted a dog named Ollie. A visitor comes by for a late lunch and reaches toward Ollie to introduce themselves. Ollie stiffens as he pulls back and growls at the visitor. The interaction lasts only a few seconds, and the visitor backs away.
High-intensity	You are having a visitor over for breakfast one morning. You and your visitor are sitting at the kitchen island when your newly adopted cat, Charlie, steps into the entryway of the kitchen. Upon seeing your visitor, Charlie’s ears pin back as he hisses, then he slowly walks up to the visitor’s chair. Charlie then hisses again and bares his teeth at the visitor’s leg. Charlie yowls and stays close to the visitor.	On a Friday evening, you decide to invite a visitor over for a movie night. You are both sitting on the couch when your newly adopted dog, Max, comes through the hallway. Upon seeing your visitor, Max pins his ears back as he growls, then he continues his way into the living room. Max approaches the visitor and growls again, then bares his teeth at the visitor’s leg. Max barks and keeps a close eye on the visitor.

The between-subjects factor was intensity of defensive behavior. The within-subjects factor was species.

#### Participant tasks

##### Emotion rating

Participants were given the following instructions: “On a scale of 1 (*not at all*) to 5 (*very*), please answer the following questions based on the above scenario. There are no right or wrong answers.” To increase the robustness of the measures, two items were averaged together to compute each emotion score. Thus, a fear score was obtained by averaging the scores of questions “How anxious do you think Charlie feels?” and “How afraid do you think Charlie feels?” The same technique was utilized for the anger score, with questions “How angry do you think Charlie feels?” and “How annoyed do you think Charlie feels?” As such, each participant rated both perceived fear and perceived anger for each vignette. Fear and anger were selected because they are the most common emotions underlying defensive or problem behaviors and are frequently mistaken for one another (Bowen and Heath, 2005; Guo et al., 2024). The names included corresponded to the vignette each participant had seen immediately prior, and all questions were presented in randomized order.

##### Intervention technique

Participants were instructed to “Imagine that Ollie has shown this type of behavior a few times before, at least three. With that in mind, answer the following questions. There are no right or wrong answers.” They were then asked “What is the likelihood you would do each of the following.” and responded on a scale from 1 (*very unlikely*) to 4 (*very likely*). The intervention techniques included moving the companion animal to another room in the house (relocate), considering taking the companion animal back to the shelter (relinquish), reaching out to a behavioral trainer (trainer), considering medication for the companion animal (medication), attempting to find a new home for the companion animal (rehome), and spanking the companion animal (spank). Statements such as “Consider medication for Milo” and “Try and find a new home for Charlie” were used to present the intervention techniques to participants, and all statements were randomly ordered. The name of the animal included in the intervention technique instructions and questions matched the name presented in the emotion rating questions and the vignette that preceded this task.

#### Questionnaires

##### Beliefs About Animal Emotions Scale (BAES)

The 23-item Beliefs About Animal Emotions Scale (BAES; Kisley, 2025) was used to measure participants’ pre-existing beliefs about animal emotions. Instructions read “We are interested in your beliefs about the emotional lives of animals. For *animals*, you may think of pets, farm animals, wildlife, and other animals. For *emotions*, you may think of feelings like sadness, happiness, fear, and other feelings. There are no right or wrong answers. We are interested in your personal viewpoint.” Items on the BAES are divided into three subscales: Complexity and Authenticity (e.g., “The emotional world of most animals is too limited to be considered ‘complex.’”), Independence from Human Similarity (e.g., “A monkey feels a richer range of emotions than a lizard”), and Moral Relevance of Animal Emotions (e.g., “Animal emotions are less important than human emotions.”). Items are rated on a 7-point Likert-type scale ranging from 1 *strongly agree* to 7 *strongly disagree*. In general, stronger disagreement with these items indicates a stronger belief that animals experience emotions and have the capacity for emotional experience. Two items in the scale were reverse coded to a scale of 1 (*Strongly agree*) to 7 (*Strongly disagree*), specifically in the Moral Relevance subscale. All items were presented to participants in random order. Items were averaged to obtain a score for each subscale, with higher scores indicating stronger beliefs about animal emotions. The Complexity and Authenticity subscale originally had a Cronbach’s α of 0.95, while in the current study, Cronbach’s α was 0.94. The Independence of Participants subscale originally had a Cronbach’s α of 0.84, while in the current study, Cronbach’s α was 0.94. The Moral Relevance subscale originally had a Cronbach’s α of 0.94, while in the current study, Cronbach’s α was 0.95.

##### Implicit Theories of Emotion Scale

We also included an adapted scale to assess whether participants’ beliefs about pets’ ability to control or change their emotions would be predictive of intervention approaches. The rationale for this was that people who believe that pets can control their experiences of fear or anger may be less likely to intervene. For this purpose, and following the general approach of previous studies that have modified this scale by changing the referent for all items (De Castella et al., 2013; Shulkin et al., 2025), we adapted Tamir et al.’s (2007) Implicit Theories of Emotion Scale to refer to animals rather than “everyone” or “people.” “Animals can learn to control their emotions” and “If they want to, animals can change the emotions that they have” represent so-called “incremental” beliefs, that is, the belief that emotions can be controlled. “No matter how hard they try, animals cannot really change the emotions that they have” and “The truth is, animals have very little control over their emotions” were intended to represent “entity” beliefs. Participants were instructed to “Please carefully read the following questions and select the answer you agree most with. There are no right or wrong answers. For animals, you may think of pets, farm animals, wildlife, and other animals. For emotions, you may think of feelings like sadness, happiness, fear, and other feelings.” They answered on a 4-point scale ranging from 1 (*Strongly disagree*) to 4 (*Strongly agree*). All items were randomly presented to participants. The entity items were reverse-coded, and all four items were averaged to produce a single score for each participant, where “higher scores indicate incremental theories and lower scores indicate entity theories of emotion” (Tamir et al., 2007). After reverse-scoring the entity items and forming a composite variable of the Implicit Theories of Emotion Scale, Tamir et al. reported a Cronbach’s α of 0.75, and the adapted scale used in the present study showed a Cronbach’s α of 0.91.

##### Experience Scales

To measure participants’ experience with dogs, the *Expertise* measure developed by Kujala et al. (2017) was implemented, and this measure was also adapted to assess experiences with cats. Participants were given the instructions to “Please answer the following questions to best represent you and your experiences.” Statements such as “How much do you know about dog [cat] behavior?,” “How experienced are you in identifying dog [cat] behavior?,” and “How much do you like dogs [cats]?” were included for each species in each scale. Participants answered on a sliding scale ranging from 0 to 100, and all items were randomly presented to participants. After data collection, composite scores for both dog and cat experience were computed by calculating the mean of the items for each scale. Cronbach’s α was not reported by Kujala et al.; however, it was assessed for both the dog and cat experience scales in the present study, yielding a Cronbach’s α of 0.92 and 0.91, respectively.

In the cat experience scale, the final question was changed from “How much are you interested about animal behavior in general?” to “How much are you interested about human behavior in general?” to ensure that the final question asked in the dog experience scale was not duplicated. Scored responses measuring interest in human behavior were then replaced with participants’ scores for interest in animal behavior in general. This adjustment was performed to ensure that the cat experience composite score used the same operationalization of animal behavior as the composite score for dog experience.

##### Responsibility Scales

Similar to the adapted *Expertise* measure mentioned above, Kujala et al.’s (2017) *Exposure* measure was used in the current study and adapted to refer to cats separately. Only one item from the *Exposure* measure was utilized in the statistical analysis: “Have you been responsible for a dog[cat] in your family? If yes, for how many years have you been responsible for a dog/dogs[cat/cats] in your family?” The response options were as follows: 1 (*Not at all*), 2 (*Under 1 year*), 3 (*1–5 years*), 4 (*6–10 years*), and 5 (*Over 10 years*). This item was selected because it assesses a person’s direct experience caring for a companion animal. It was analyzed separately from the other items on the *Exposure* measure because they were not all rated on the same scale.

### Procedure

After reviewing a brief study description on Prolific, participants were redirected to Qualtrics, where they provided click-through informed consent and completed the survey, which was expected to take approximately 15 min. Next, participants were block-randomly assigned to either the low-intensity or high-intensity condition. The vignettes were presented to participants in random order. Each participant read either the dog or the cat vignette, followed by the emotion-rating task and the intervention technique task. After reading the first vignette and completing the subsequent tasks, participants repeated the process for the remaining vignette. Participants in the low-intensity condition were presented only with vignettes depicting low-intensity behavior, whereas those in the high-intensity condition were presented only with vignettes depicting high-intensity behavior. After completing the tasks for both species, participants completed the questionnaires and subsequently provided demographic information, including age, sex, ethnicity, and the highest level of education completed. Finally, participants were redirected to Prolific to complete their submission.

### Statistical analysis

All statistical analyses were performed using IBM SPSS Statistics version 31.0.0.0. A mixed-design ANOVA was performed to investigate differences in the emotion perceived by participants across conditions (between-subjects) and species (within-subjects). A second mixed-design ANOVA was performed to examine differences in selected intervention techniques across conditions (between-subjects) and species (within-subjects). To control for multiple comparisons, Bonferroni adjustment was applied in SPSS to the *post hoc* comparisons used to examine the direction of significant effects in the repeated measures ANOVAs performed.

Multiple linear regressions were performed separately for each intervention technique for dogs and cats. For each multiple linear regression, the following variables were included as predictors: experimental condition (intensity of defensive act), perceived anger and fear scores, participants’ BAES Moral Relevance of Animal Emotions and BAES Complexity and Authenticity subscale scores (Kisley, 2025), Implicit Theories of Emotion Scale scores, exposure scores, and the responsibility item from the exposure scale (Kujala et al., 2017). The experience score and responsibility item were included to examine how experience with each animal affects one’s likelihood of utilizing each intervention technique, as these factors have been shown to affect the accuracy of interspecific emotional perception (de Mouzon et al., 2023; Molinaro and Wynne, 2025). Experimental condition, fear scores, and anger scores were all included to investigate how the intensity of the behavior and the inferred underlying emotion may affect how people interact with and intervene in situations involving companion animals. The Moral Relevance of Animal Emotions and Complexity and Authenticity subscales of the BAES were included because beliefs about animal emotions have been linked to attitudes toward animals and objective animal welfare (Haddy et al., 2025; Hancock et al., 2023; Tamioso et al., 2018). Finally, Implicit Theories of Emotion Scale scores were included to assess whether beliefs about the controllability of animal emotions predict human intervention techniques.

## Results

### Primary findings

#### Emotion perception

**Figure 1:**
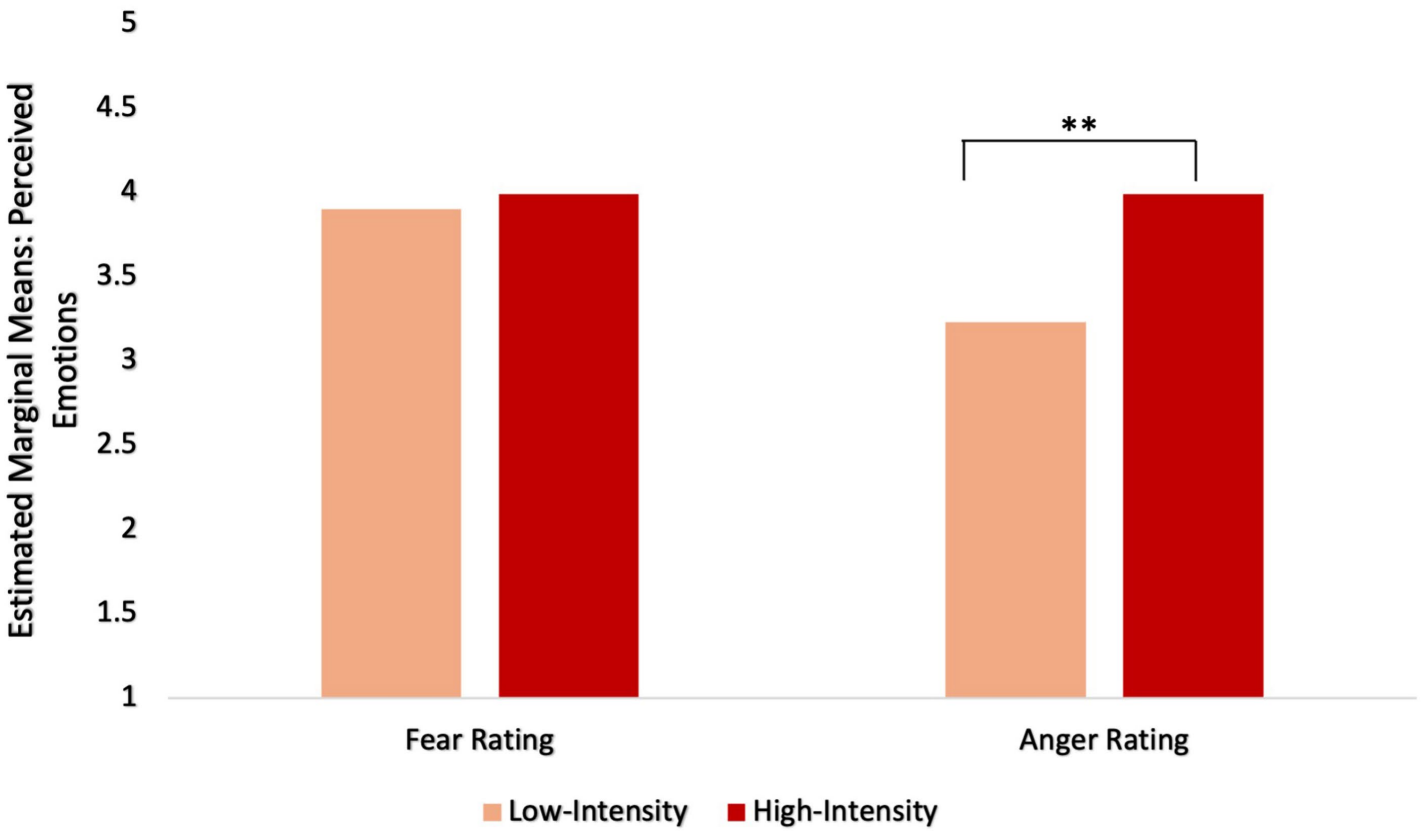
Emotion type x experimental condition interaction effect. Main effects are not indicated in the graph; only the interaction effect between emotion type and experimental condition is shown. **p < 0.001.

A mixed-design ANOVA was performed to examine the effects of species type and intensity of defensive behaviors on perceived fear and anger. Pairwise comparisons were performed to assess the direction of any significant findings. Descriptive statistics for emotion scores are shown in Table 2 (top). A main effect of experimental condition was found, *F*(1, 192) = 17.71, *p* < 0.001, η^2^_p_ = 0.08. This effect was observed such that participants perceived higher emotion overall in the high-intensity condition (*EMM =* 3.99, *SE* = 0.07) compared to the low-intensity condition (*EMM* = 3.56, *SE* = 0.07). A main effect of species was also found, *F*(1, 192) = 4.10, *p* < 0.05, η^2^_p_ = 0.02. When reading the cat vignette (*EMM* = 3.82, *SE* = 0.06), participants rated significantly higher levels of emotion compared to the dog vignette (*EMM* = 3.73, *SE* = 0.05). A main effect of emotion type was found, *F*(1, 192) = 22.06, *p* < 0.001, η^2^_p_ = 0.10. Participants were more likely to attribute fear (*EMM* = 3.94, *SE* = 0.06) to the companion animals compared to anger (*EMM* = 3.61, *SE* = 0.06) across intensity conditions. An interaction effect was found between emotion type and experimental condition, *F*(1, 192) = 21.72, *p* < 0.001, η^2^_p_ = 0.10. The perception of fear underlying the companion animal’s defensive act did not significantly differ between participants in the low-intensity condition (*EMM* = 3.90, *SE* = 0.09) and those in the high-intensity condition (*EMM* = 3.99, *SE* = 0.09). In contrast, the perception of anger in the companion animals was significantly lower in the low-intensity condition (*EMM* = 3.23, *SE* = 0.09) compared to the high-intensity condition (*EMM =* 3.99, *SE* = 0.09), as shown in Figure 1.

**Table 2:** Descriptive statistics of emotional perception and intervention technique use across conditions.

Reported variables	Low-intensity		High-intensity	
	Cat	Dog	Cat	Dog
	*M* (*SD*)	*M* (*SD*)	*M* (*SD*)	*M* (*SD*)
Emotion				
Fear	3.91 (1.03)	3.88 (0.96)	4.00 (0.86)	3.98 (0.87)
Anger	3.29 (1.15)	3.16 (1.09)	4.07 (0.84)	3.91 (0.82)
Intervention technique				
Relocate	2.66 (1.12)	2.79 (1.08)	3.46 (0.76)	3.40 (0.82)
Relinquish	1.40 (0.81)	1.46 (0.84)	1.36 (0.71)	1.47 (0.79)
Train	2.38 (1.16)	2.89 (1.08)	2.46 (1.07)	3.05 (1.01)
Medicate	1.63 (0.94)	1.81 (0.94)	1.89 (1.00)	2.06 (1.04)
Rehome	1.37 (0.73)	1.43 (0.76)	1.35 (0.71)	1.45 (0.79)
Spank	1.16 (0.49)	1.21 (0.59)	1.21 (0.58)	1.30 (0.68)

Levels of emotion perceived were measured on a 5-point Likert-type scale from 1 (*Not at all*) to 5 (*Very*). Likelihood of utilizing intervention techniques was measured on a 4-point Likert-type scale from 1 (*Very unlikely*) to 4 (*Very likely*).

#### Intervention technique

**Figure 2:**
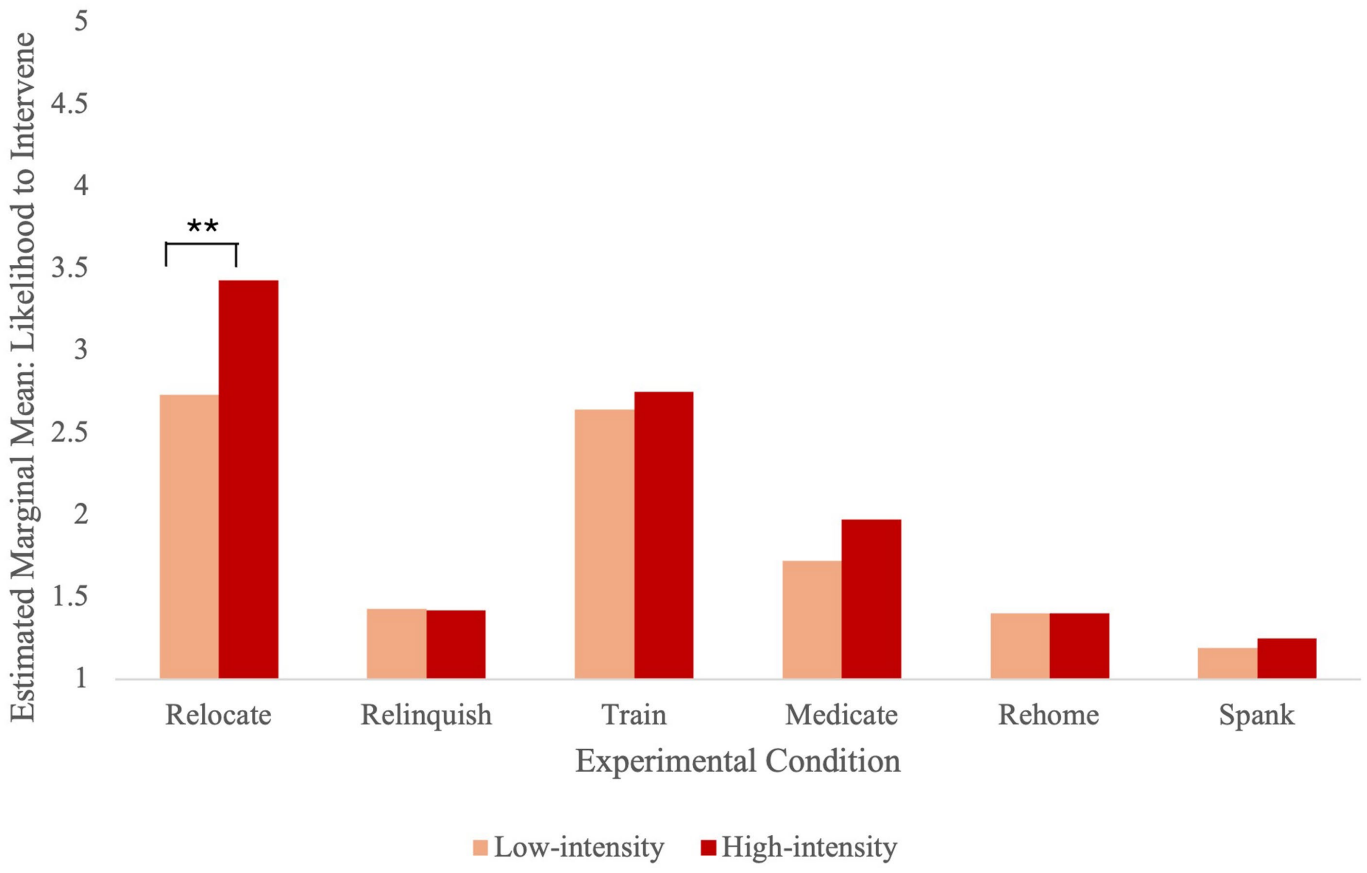
Intervention type × experimental condition interaction effect. Main effects are not indicated in the graph; only the interaction effect between intervention type and experimental condition is shown. **p < 0.001.

**Figure 3:**
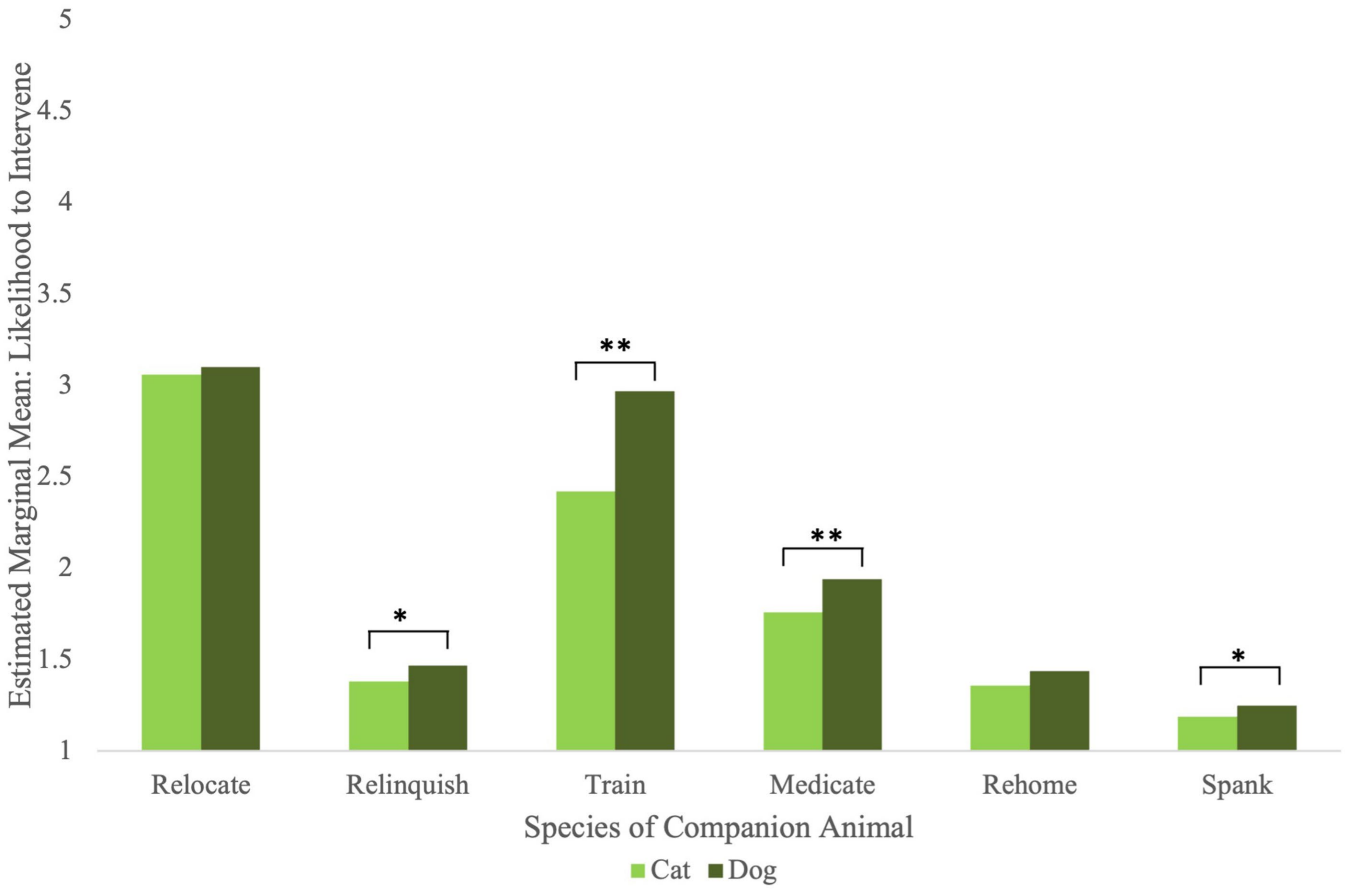
Species type × intervention type interaction effect. Main effects are not indicated in the graph; only the interaction effect between intervention type and species type is shown. *p < 0.05, **p < 0.001.

A mixed-design ANOVA was performed to examine the effects of species type and intensity of defensive behaviors on the likelihood of using one of six intervention techniques. Pairwise comparisons were performed to assess the direction of any significant findings. Descriptive statistics for intervention technique scores are shown in Table 2 (bottom). A between-subjects main effect of experimental condition was found, *F*(1, 192) = 7.91, *p* < 0.05, η^2^_p_ = 0.04. Participants in the high-intensity condition (*EMM* = 2.04, *SE* = 0.05) were significantly more likely to intervene compared to those in the low-intensity condition (*EMM* = 1.85, *SE* = 0.05). A main effect of species was found, such that participants were significantly more likely to intervene with dogs (*EMM* = 2.03, *SE* = 0.04) compared to cats (*EMM* = 1.86, *SE* = 0.04), *F*(1, 192) = 47.70, *p* < 0.001. A main effect of intervention technique was found, *F*(1, 192) = 217.32, *p* < 0.001, η^2^_p_ = 0.53. Follow-up tests revealed that participants were more willing to intervene by relocating the companion animal (*EMM* = 3.08, *SE* = 0.06) compared to all other intervention techniques (*EMM_relinquish_ =* 1.43, *SE_relinquish_* = 0.05; *EMM_trainer_ =* 2.70, *SE_trainer_ = 0.07; EMM_medicate_ =* 1.85, *SE_medicate_* = 0.07; *EMM_rehome_ =* 1.40, *SE_rehome_* = 0.05; *EMM_spank_ =* 1.22, *SE_spank_* = 0.04). A significant interaction effect between intervention type and experimental condition was found, *F*(5, 960) = 6.78, *p* < 0.001, η^2^_p_ = 0.03, as illustrated in Figure 2. Participants were significantly more likely to bring the animal to another room in the high-intensity condition (*EMM* = 3.43, *SE* = 0.09) compared to the low-intensity condition (*EMM* = 2.73, *SE* = 0.09), whereas none of the other intervention techniques significantly differed in likelihood ratings across experimental conditions (all *p*s > 0.05). An significant interaction effect between intervention type and species was also found, *F*(5, 960) = 19.42, *p* < 0.001, η^2^_p_ = 0.09. Pairwise comparisons illustrated that participants were significantly more likely to relinquish a dog (*M* = 1.47) compared to a cat (*M* = 1.38), *p* < 0.05; to take a dog (*M* = 2.97) to a trainer compared to a cat (*M* = 2.42), *p* < 0.001; to put a dog (*M* = 1.94) on medication compared to a cat (*M* = 1.76), *p* < 0.001; and to spank a dog (*M* = 1.25) compared to a cat (*M* = 1.19), *p* < 0.05, as shown in Figure 3.

#### Predictors of intervention technique—cats

The results of the regression analyses conducted to determine predictors of intervention techniques for cats are shown in the top half of Table 3 and are described in more detail below.

**Table 3:** Regression model statistics for the prediction of intervention technique likelihood outcomes.

Outcome	*F*	*p*	*R^2^*	Significant individual predictors (*β*)
Cat				
Relocate	7.850	<0.001	0.253	Experimental condition (0.38), cat fear rating (0.25)
Relinquish	3.920	<0.001	0.145	BAES moral relevance (−0.24)
Train	1.402	0.198	0.057	–
Medication	2.356	0.020	0.092	BAES complexity and authenticity (−0.24)
Rehome	3.580	<0.001	0.134	BAES moral relevance (−0.19), cat experience (−0.19), cat responsibility (0.17)
Spank	4.342	<0.001	0.158	BAES moral relevance (−0.22), cat anger rating (0.22)
Dog				
Relocate	6.424	<0.001	0.217	Experimental condition (0.31), dog fear rating (0.22), dog experience (−0.16)
Relinquish	6.047	<0.001	0.207	BAES moral relevance (−0.32), dog anger rating (0.26)
Train	1.965	0.053	0.078	–
Medication	4.594	<0.001	0.166	Dog experience (−0.29)
Rehome	2.875	0.005	0.111	Dog anger rating (0.19)
Spank	3.866	<0.001	0.143	None

*N* = 194. Each of the six intervention techniques was regressed on individual perspective and perception variables (fear and anger ratings, BAES subscale scores, experience and responsibility, and experimental condition). Significant individual predictors are standardized *β*-weights and are shown only when full regression models were significant (*p* < 0.05). All predictors listed above are significant at least at *p* < 0.05.

##### Relocating

The overall regression model for relocating one’s cat to another room was significant, *F*(8, 185) = 7.85, *p* < 0.001, with the predictors accounting for 25% of the variance (*R^2^* = 0.253). Experimental condition was a positive predictor, *B* = 0.77, *SE* = 0.41, β = 0.38, *t*(185) = 5.41, *p* < 0.001, and perceived fear was also a positive predictor of relocating one’s cat to another room in the house, *B* = 0.27, *SE* = 0.07, β = 0.25, *t*(185) = 3.67, *p* < 0.001.

##### Relinquishing

The overall model for relinquishing one’s cat to a shelter was significant, *F*(8, 185) = 3.92, *p* < 0.001, with the predictors accounting for 14% of the variance (*R^2^* = 0.145). Beliefs about the moral relevance of animal emotions were the only significant predictor, in a negative direction, *B* = −0.12, *SE* = 0.05, β = −0.24, *t*(185) = −2.55, *p* < 0.05, although two predictors were trending toward significance: perceived anger of the cat, which was a positive predictor, *B* = 0.11, *SE* = 0.06, β = 0.15, *t*(185) = 1.94, *p* = 0.054, and one’s experience with a cat, which was a negative predictor, *B* = −0.004, *SE* = 0.002, β = −0.14, *t*(185) = 1.93, *p* = 0.055.

##### Trainer

The overall model for pursuing a trainer for one’s cat was not significant, *F*(8, 185) = 1.40, *p* = 0.198, *R^2^ = 0*.057.

##### Medication

The overall model for using medication for one’s cat was significant, *F*(8, 185) = 2.36, *p* < 0.05, with the predictors accounting for over 9% of the variance (*R*^2^ = 0.092). Beliefs about the complexity and authenticity of animal emotions were the only significant negative predictor, *B* = −0.21, *SE* = 0.09, β = −0.24, *t*(185) = −2.45, *p* < 0.05.

##### Rehoming

The overall model for rehoming one’s cat was significant, *F*(8, 185) = 3.58, *p* < 0.001, with the predictors accounting for 13% of the variance (*R*^2^ = 0.134). Many predictors emerged as significant. Beliefs about the moral relevance of animal emotions were a significant negative predictor, *B* = −0.09, *SE* = 0.04, β = −0.19, *t*(185) = −2.02, *p* < 0.05, as was one’s experience with cats, *B* = −0.01, *SE* = 0.002, β = −0.19, *t*(185) = −2.54, *p* < 0.05. One’s responsibility for a cat was a significant positive predictor, *B* = 0.08, *SE* = 0.04, β = 0.17, *t*(185) = 2.39, *p* < 0.05.

##### Spanking

The overall regression model for spanking one’s cat was significant, *F*(8, 185) = 4.34, *p* < 0.001, with the predictors accounting for nearly 16% of the variance (*R^2^* = 0.158). Beliefs about the moral relevance of animal emotions were a significant negative predictor, *B* = −0.08, *SE* = 0.03, β = −0.22, *t*(185) = −2.36, *p* < 0.05, and perceived anger of the cat was a significant positive predictor, *B* = 0.11, *SE* = 0.04, β = 0.22, *t*(185) = 2.89, *p* < 0.05.

#### Predictors of intervention technique—dogs

The results of the regression analyses conducted to determine predictors of intervention techniques for dogs are shown in the bottom half of Table 3 and are described in more detail below.

##### Relocating

The overall regression model for relocating one’s dog to another room was significant, *F*(8, 185) = 6.42, *p* < 0.001, with the predictors accounting for 22% of the variance (*R*^2^ = 0.217). Experimental condition was a significant positive predictor, *B* = 0.62, *SE* = 0.14, β = 0.31, *t*(185) = 4.37, *p* < 0.001, and dog’s perceived fear was a positive predictor of moving one’s dog to another room in the house, *B* = 0.24, *SE* = 0.08, β = 0.22, *t*(185) = 3.14, *p* < 0.05. Experience with dogs, however, was a negative predictor, *B* = −0.01, *SE* = 0.003, β = −0.16, *t*(185) = −2.17, *p* < 0.05. Finally, beliefs about the complexity and authenticity of animal emotions were trending toward significance as a negative predictor, *B* = −0.17, *SE* = 0.08, β = −0.19, *t*(185) = −1.97, *p* = 0.050.

##### Relinquishing

The overall model for relinquishing one’s dog to a shelter was significant, *F*(8, 185) = 6.05, *p* < 0.001, with the predictors accounting for almost 21% of the variance (*R*^2^ = 0.207). Beliefs about the moral relevance of animal emotions were a significant negative predictor, *B* = −0.17, *SE* = 0.05, β = −0.32, *t*(185) = −3.44, *p* < 0.001, and perceived anger of the dog was a positive predictor, *B* = 0.20, *SE* = 0.06, β = 0.26, *t*(185) = 3.52, *p* < 0.001.

##### Trainer

The overall model for pursuing a trainer for one’s dog was trending toward significance, *F*(8, 185) = 1.97, *p* = 0.053, *R*^2^ *= 0*.078.

##### Medication

The overall model for using medication for one’s dog was significant, *F*(8, 185) = 4.59, *p* < 0.001, with the predictors accounting for nearly 17% of the variance (*R*^2^ = 0.166). Experience with dogs was the only significant predictor, in a negative direction, *B* = −0.01, *SE* = 0.003, β = −0.29, *t*(185) = −3.71, *p* < 0.001.

##### Rehoming

The overall model for rehoming one’s dog was significant, *F*(8, 185) = 2.88, *p* < 0.05, with the predictors accounting for 11% of the variance (*R*^2^ = 0.111). Perceived anger of the dog was a significant positive predictor, *B* = 0.15, *SE* = 0.06, β = 0.19, *t*(185) = 2.50, *p* < 0.05.

##### Spanking

The overall regression model for spanking one’s dog was significant, *F*(8, 185) = 3.87, *p* < 0.001, with the predictors accounting for over 14% of the variance (*R*^2^ = 0.143). None of the predictors were significant, while beliefs about the moral relevance of animal emotions were trending toward significance as a negative predictor, *B* = −0.08, *SE* = 0.04, β = −0.19, *t*(185) = −1.93, *p* = 0.055.

## Discussion

The findings of the current study suggest that intervention techniques used in response to companion animal defensive behaviors are related to many factors. These include participant characteristics such as experience with pets and beliefs about and perceptions of animal emotions, as well as external factors, such as the species involved and the intensity of the behavioral display. Overall, the results suggest that human responses to challenging animal behavior reflect both features of the situation and differences in how people interpret the animal’s underlying emotional state. The present study extends previous research on the perception of animal emotions by linking those perceptions and beliefs to intended intervention strategies. The primary findings are examined more closely below.

### Primary findings

Building on prior research showing that between-subjects factors are meaningful in explaining variation in how people interpret animal behavior and perceive underlying emotions, the present study examined whether such differences were also associated with individuals’ intended intervention strategies. Among the belief variables, beliefs about the moral relevance of animal emotions emerged as especially important. In response to cat defensive behavior, these beliefs negatively predicted relinquishment to a shelter, rehoming, and spanking, and they also negatively predicted relinquishing a dog to a shelter. This pattern suggests that viewing animal emotions as more morally important may function as a protective factor against some higher-impact responses. Participants’ perceptions of the animal’s emotion also mattered. Higher perceived fear in both cats and dogs positively predicted relocating the animal, suggesting that this relatively low-impact response is more likely when individuals interpret the behavior as fear-based. Higher perceived anger, by contrast, positively predicted spanking toward cats and relinquishment and rehoming of dogs, indicating that interpreting defensive behavior as anger may be associated with harsher or more disruptive interventions. Experience with and responsibility for pets showed a smaller, more species-specific pattern: experience with dogs negatively predicted relocation and medication for dogs, whereas experience with cats negatively predicted rehoming a cat, and responsibility for cats positively predicted rehoming. Taken together, these findings extend prior research on the connection between participant characteristics and perceptions of animal emotions by showing that such differences are associated not only with how people understand companion animals (Amici et al., 2019; Lakestani et al., 2014; Aldridge and Rose, 2019; Wan et al., 2012; Catalán et al., 2020; Guo et al., 2024; Barklam and Felisberti, 2024) but also with how they say they would respond to challenging behavior.

The companion animal species involved also played an important role in both emotional perception and intended intervention. In the current study, participants rated slightly higher overall levels of emotion when reading cat scenarios compared to dog scenarios. This is notable, as prior research using dog and cat vocalizations has found greater perceived emotionality in dogs than in cats, suggesting that the form of interspecific communication or stimulus modality may influence how people perceive animal emotions (Barklam and Felisberti, 2024). Consistent with this possibility, previous research has shown that the accuracy of attributing animal emotions varies depending on how the stimulus is presented, with video and audio together generally supporting more accurate judgments than either form of presentation alone (de Mouzon et al., 2023). At the same time, participants in the present study were overall more willing to intervene with dogs than with cats, although this species difference varied by intervention type. Specifically, participants were more likely to report that they would relinquish a dog, bring a dog to a trainer, use medication for a dog, or spank a dog than to endorse the same responses for a cat. Taken together, these findings suggest that emotional perception and intended intervention do not map onto species in a simple one-to-one manner: cats were perceived as slightly more emotional overall, yet dogs elicited more intervention, particularly for several of the more consequential responses. This pattern highlights the importance of species-specific expectations regarding how companion animal behavior is interpreted and how people would likely respond to it.

In the present study, the intensity of defensive behavior shaped both emotional perception and intended intervention. Participants perceived higher overall levels of emotion in the high-intensity condition than in the low-intensity condition, suggesting that even within a written vignette, the severity of a behavioral display influences how strongly people perceive an underlying emotional state. This expected pattern also serves, to some extent, as a manipulation check, indicating that the vignettes successfully varied perceived behavioral intensity. The finding aligns with prior research showing that contextual features of a stimulus can influence how people perceive animal emotions (Molinaro and Wynne, 2025; Guo et al., 2024). The pattern differed between anger and fear. Overall, participants attributed more fear than anger to the animals, but anger differentiated the high- and low-intensity conditions. In other words, low-intensity defensive behavior was interpreted primarily in terms of fear, whereas high-intensity behavior increased perceptions of anger, such that fear and anger were rated at similar levels. This is consistent with previous research showing that fear and anger are often confused in the interpretation of defensive behavior, while also suggesting that increases in behavioral intensity may lead people to interpret the animal as more hostile rather than simply more distressed (Bowen and Heath, 2005; Bouma et al., 2023; Guo et al., 2024). Intensity was also related to intended intervention, as participants were overall more likely to endorse intervening in the high-intensity condition than in the low-intensity condition. This expected difference further suggests that the vignettes successfully manipulated the severity of the behavioral display. Together, these findings suggest that variation in behavioral intensity shapes both emotional interpretation and people’s intended responses to companion animal defensive behavior.

### Practical implications

To the best of our knowledge, the present study is among the first to examine how people report their likelihood of using different intervention techniques in response to defensive behavior in companion animals. Across intensity conditions and species, relocating the animal to another room was preferred over all other intervention types. This suggests that when faced with unwanted defensive behavior, people appear to favor an intervention that can be implemented immediately and at relatively low cost, especially compared to options such as using medication, consulting a trainer, rehoming, or relinquishment. These findings highlight the importance of understanding what leads people to prefer one response over another, as intervention strategies are not equivalent in their likely consequences for the human–animal relationship or for the animal’s experience (Dybdall et al., 2007; Guilherme Fernandes et al., 2017; Vieira de Castro et al., 2020; Argüelles et al., 2023; Lopes et al., 2022). Although the present study did not assess the actual outcomes of these responses, some interventions are clearly more disruptive or aversive than others, suggesting the value of helping companion animal guardians make more informed choices when responding to defensive behavior (Guilherme Fernandes et al., 2017; Vieira de Castro et al., 2020). The present results indicate that how people perceive and interpret an animal’s emotional state is related to the kinds of corrective actions they say they would consider, suggesting that a better understanding of animal emotions could support more constructive responses to challenging behavior. Teaching companion animal guardians about animal emotions, including but not limited to how to interpret defensive behavior through the lens of emotion, may be a useful complement to behavior-management efforts (Fear Free, 2016; Wauthier et al., 2025).

### Study limitations

Several limitations should be considered when interpreting the present findings. Most notably, the outcome measures were based on self-reported likelihood of using different intervention techniques rather than on observed behavior. Accordingly, participants’ responses may not fully reflect what they would actually do when confronted with a defensive companion animal in real life, particularly for socially undesirable responses such as spanking or relinquishment. A related issue is that beliefs about emotions may not always be fully explicit or available to conscious awareness (Connors and Halligan, 2015; Kisley et al., 2024b), meaning that self-report measures may not capture all belief-based processes influencing how people interpret animal behavior and decide how to respond. In addition, one of the variables used to represent prior responsibility for dogs and cats was based on a single item rather than a broader composite measure (adapted from Kujala et al., 2017), which likely limited the precision with which that construct was assessed. Future research would therefore benefit from combining self-report measures with more behaviorally anchored outcomes and from using stronger multi-item assessments of important between-subjects variables such as responsibility for companion animals.

Another potential limitation concerns the use of written vignettes to represent defensive behavior in cats and dogs. This approach offered important advantages in standardization and experimental control, but it also simplified the multimodal cues people encounter in real interactions with companion animals. In everyday settings, emotional interpretation is likely shaped not only by a description of behavior but also by visual signals, vocalizations, environmental cues, and the real-time dynamics of the interaction. Therefore, although the present findings suggest that even written descriptions of defensive behavior can affect emotional perception and intended intervention, future research should examine whether similar patterns emerge when participants respond to video, audio, or more naturalistic behavioral displays. In addition, because the written vignettes described the companion cats and dogs as “recently adopted,” it is possible that participants envisioned the companion animals at many different ages ranging from very young to very old; this possible animal age difference could also have influenced participants’ subsequent intervention behavior. While the effect of animal age on intervention behavior has not yet been studied, evidence does suggest that younger faces (for both humans and companion animals) are associated with stronger motivation for caregiving behaviors compared to older faces (Borgi and Cirulli, 2016). Therefore, it is plausible that the perceived age of a companion animal could influence human corrective behavior following that animal’s defensive act. Despite these limitations, the present use of vignettes helps broaden the methodological scope of this literature, which is only just beginning to examine how different forms of stimulus presentation influence perceptions of animal emotion, rather than accuracy alone (de Mouzon et al., 2023).

A further limitation concerns the measurement of perceived emotion. As planned, the fear score was calculated by averaging ratings of “afraid” and “anxious,” whereas the anger score was calculated by averaging “angry” and “annoyed” ratings. Although the paired items were intended to capture closely related emotional states in both cases, the individual terms are not necessarily interchangeable. Averaging them may have therefore obscured potentially meaningful distinctions in how participants interpreted the animals’ behavior. *Post hoc*, we decided to investigate this. Item-level descriptive statistics are provided in Supplementary Table S1. For the anger-based items, ratings of “angry” and “annoyed” showed similar descriptive patterns across species and intensity conditions, supporting their use as indicators of a broader anger-related appraisal. For the fear-related items, ratings of “afraid” and “anxious” were both relatively high across conditions and similar across species, but they differed descriptively in their relation to behavioral intensity: anxious ratings were higher in the high-intensity condition, whereas afraid ratings remained relatively stable. As the study was designed and analyzed using only composite scores, we did not conduct inferential analyses of the individual items, so these descriptive patterns should be interpreted cautiously. Overall, however, these observations provide evidence that the composite scores appear to provide useful broad indicators of perceived fear-related and anger-related emotion as intended, although future research may benefit from distinguishing more precisely among related emotion terms.

Similarly, other measures used here may also introduce potential limitations. For example, some items in the scale developed by Kujala et al. (2017) to assess experience with animals have relatively clear face validity, such as “How much do you know about dog [cat] behavior?” and “How experienced are you in identifying dog [cat] behavior?” However, other items, such as “How much do you like dogs [cats]?” and “How much are you interested about dogs [cats]?”, may appear to better represent general affinity or liking toward the two species, instead of clearly assessing experience with animals. Post hoc, we examined potential differences in ratings based on apparent face validity. Supplementary Table S2 presents the descriptive statistics for all items for both species. Indeed, the items referring to liking and interest exhibited somewhat higher values than those referring explicitly to experience with animals. As the study was designed and analyzed using only composite scores, these *post hoc* descriptive values for individual items should be interpreted cautiously. However, it is worth noting that the scores for what we treated as experience with animals here may reflect liking/interest in animals as much as experience. Either way, we consider the variation in apparent face validity within the scale to be acceptable, as Cronbach’s alpha for both the cat and dog versions of the scale was in the excellent range. Unfortunately, we did not ask participants specifically about their past *professional* experience with animals, although this is known to impact perceptions of animal emotions and interpretations of animal behavior (Catalán et al., 2020). Finally, it is important to note that the adapted version of the Implicit Theories of Emotion Scale (Tamir et al., 2007) used here has not been validated for use with the referent “animals,” instead of the original referents “everyone” and “people.” As such, it would be premature to conclude that the lack of significant findings for this adapted scale implies that human beliefs about the relative controllability of animal emotions are not relevant to interpretations of pet behavior. In our view, future research with more rigorously developed and validated scales that distinguish between incremental and entity belief frameworks is still warranted.

## Conclusion

Taken together, the findings of the present study highlight the importance of human perceptions and beliefs about animal emotions in shaping responses to defensive behavior in companion animals. The results suggest that how people interpret an animal’s behavior—particularly in terms of fear, anger, and the moral relevance of animal emotions—is importantly associated with the kinds of corrective actions they say they would consider. More broadly, this study extends previous research on the perception of animal emotions by linking those perceptions and beliefs to intended intervention strategies in a practical companion animal context. Understanding these processes may help inform future efforts to support companion animal guardians in responding more constructively to challenging behavior and may, in turn, contribute to more positive human–animal relationships.

## Data Availability

The datasets presented in this study can be found in online repositories. The names of the repository/repositories and accession number(s) can be found below: https://osf.io/fxsbq.
