# Supplementary material for: Reading between the whines: human perceptions and beliefs about animal emotions predict how people would intervene with cats and dogs showing challenging behaviors
Source: Front Psychol. 2026 Jul 15;17:1857104. doi: 10.3389/fpsyg.2026.1857104 (PMC13414966; doi:10.3389/fpsyg.2026.1857104)
Supplement: Supplementary file 1 [file Table_1.docx]

**Supplementary Table 1**

*Descriptive Statistics of Perceived Animal Emotions Before and After Forming Composite Variables*

| **Emotion** | **Cat** | **Dog** |
| --- | --- | --- |
|  | *M* (*SD*) | *M* (*SD*) |
| Afraid | 3.72 (1.14) | 3.64 (1.12) |
| Anxious | 4.19 (1.00) | 4.22 (0.98) |
| **Composite:** Fear | 3.96 (0.95) | 3.93 (0.92) |
| Angry | 3.54 (1.21) | 3.44 (1.18) |
| Annoyed | 3.82 (1.15) | 3.63 (1.12) |
| **Composite:** Anger | 3.68 (1.08) | 3.54 (1.03) |

*Note.* Levels of perceived emotions were measured on a 5-point Likert-type scale from 1 (*Not at all*) to 5 (*Very*).
