# Supplementary material for: Reading between the whines: human perceptions and beliefs about animal emotions predict how people would intervene with cats and dogs showing challenging behaviors
Source: Front Psychol. 2026 Jul 15;17:1857104. doi: 10.3389/fpsyg.2026.1857104 (PMC13414966; doi:10.3389/fpsyg.2026.1857104)
Supplement: Supplementary file 2 [file Table_2.docx]

**Supplementary Table 2**

*Descriptive Statistics of Items Within Cat and Dog Experience Measures*

| **Experience Scale Items** | **Cat** | **Dog** |
| --- | --- | --- |
|  | *M* (*SD*) | *M* (*SD*) |
| How much do you like ___s? | 74.22 (30.15) | 83.60 (24.56) |
| How much are you interested about ___s? | 66.88 (33.19) | 77.86 (27.14) |
| How much do you know about ___ behavior? | 55.23 (30.94) | 64.56 (27.61) |
| How experienced are you in identifying ___ behavior? | 54.26 (32.71) | 60.65 (27.61) |
| How much are you interested about animal behavior in general? | 73.04 (25.78) | 73.04 (25.78) |
| **Composite** | 64.72 (26.37) | 71.94 (23.53) |
|  |  |  |

*Note.* Items were measured on a scale from 0 to 100.
